# Utilization and Effectiveness of Home Treatment for People With Acute Severe Mental Illness: A Propensity-Score Matching Analysis of 19 Months of Observation

**DOI:** 10.3389/fpsyt.2018.00495

**Published:** 2018-10-10

**Authors:** Sonja Mötteli, Dominik Schori, Helen Schmidt, Erich Seifritz, Matthias Jäger

**Affiliations:** ^1^Department of Psychiatry, Psychotherapy and Psychosomatics, University Hospital of Psychiatry Zurich, Zurich, Switzerland; ^2^Directorate of Nursing, Therapies and Social Work, University Hospital of Psychiatry Zurich, Zurich, Switzerland

**Keywords:** home treatment, effectiveness, evaluation, acute mental illness, propensity score matching

## Abstract

Treatment guidelines recommend home treatment (HT) as an effective alternative to inpatient treatment for individuals with severe, acute mental illness (SAMI). Nevertheless, HT is largely unfamiliar in German-speaking countries. Here we examined the utilization and effectiveness of HT services newly implemented in a large hospital setting in Switzerland. We used a naturalistic observational study design including patients (*n* = 201, 18–65 years, 65.7% females) with SAMI who received HT between June 2016 and December 2017. HT patients were compared with a crude inpatient sample (*n* = 1078) and a matched inpatient sample (*n* = 201). Propensity-score matching was used to control for personal characteristics. Treatment outcomes were compared between HT patients and the matched inpatients based on routinely obtained medical data. The results showed that the HT sample consisted of more females (+21%), older (+4 years), and better educated (+10%) patients with more affective disorders (+13%) and less substance use disorders (−15%) as compared with the crude inpatient sample. The severity of symptoms was the same. After matching, there were no significant differences in the proportion of readmissions (36%), the duration until readmission and scores of the Health of the Nation Outcome Scales (HoNOS). The treatment duration of HT patients was significantly longer and, post-treatment, scores on the Global Assessment of Functioning scale (GAF) were significantly better. We conclude that HT is an effective treatment option for patients with SAMI also in Switzerland concerning the reduction of hospital days, the improvement of symptoms and functioning and readmission rates. HT cannot fully replace hospital admissions in all cases and HT may be beneficial for particular groups of patients (e.g., females and individuals with affective disorders). The study further shows the potential value of propensity-score matching in health care service research.

## Introduction

Home treatment (HT) is an evidence-based alternative to inpatient treatment for patients with a severe, acute mental illness (SAMI) and is explicitly recommended by current treatment guidelines (1, 2). Instead of a hospital stay, patients are treated at their homes by a multidisciplinary and mobile professional team in episodes of acute mental illness (3, 4). Unlike to English-speaking (e.g., U.K., USA, Canada) and other countries such as Norway, Italy, France, and the Netherlands, HT services have not yet been widely established in German-speaking countries (5), most likely due to different cultural backgrounds and psychiatry service histories. Therefore, it is unclear whether HT is as effective as inpatient treatment in Switzerland, considering its particular financing and care structures (6).

In 2016, the University Hospital of Psychiatry Zurich, which is the largest psychiatric hospital in Switzerland, established HT services with capacity of up to 18 patients at a time. These HT services are intended as an alternative for patients with SAMI—patients with illness duration > 2 years and Global Assessment of Functioning (GAF) scores < 50 (7)—and need for inpatient treatment who are aged between 18 and 65 years, voluntarily admitted and with permanent residence in the urban and periurban surrounding of the city of Zurich. Patients can be assigned to HT by resident physicians or change to HT after initial or intermediate inpatient stays. There are no predefined exclusion criteria for any diagnoses, however, HT services are limited to patients with health insurance companies that agreed to pay for this service, which is true for about 50% of all health insurances. Moreover, patients with delirious syndromes and acute intoxications are excluded as well as acute risk of self-harm or harm to others. In case a patient meets the criteria for HT, the physician in charge discusses this treatment option with the patient considering his or her preferences and the HT occupancy rate.

The multiprofessional HT team consisting of physicians, nurses, one psychologist, one occupational therapist, and one social worker provides daily home visits, i.e., at least one (up to three) visit by nursing staff per day, and at least two consultations by physicians per week. If desired, patients can also use other hospital services (e.g., physical exercise therapy, vocational therapy, internal medicine). The team offers 24/7 accessibility.

The HT implementation is in line with the model guidelines described by Gühne et al. (8). Key characteristics of such services are mobile and multiprofessional teams, rapid access, frequent home visits, a 24-h service and the provision of different treatments, such as medical, psychological and social interventions (9). The HT team also has a “gatekeeping”—function, which aims to prevent unnecessary hospital admissions (8). The treatment duration should be similar to hospital stays (5). Although there are no strict exclusion criteria, HT is not suitable for uncooperative patients and individuals at risk of self-harm or harm to others (10). Previous research showed that HT can reduce inpatient stays and hospital admissions during acute episodes. Studies also demonstrated that HT was associated with improved cost-effectiveness, satisfaction of patients and relatives and reduced withdrawals when compared to treatment-as-usual (8, 11–13). In particular, patients with children often are interested in being treated at home (14). However, more evidence is needed concerning treatment duration, readmission rates, psychosocial outcomes, and characteristics of HT users (5).

Although randomized controlled designs are considered as “gold standard” to estimate causal treatment effects, such trials are not always feasible due to ethical or logistical reasons (15, 16). Moreover, limitations referring to external validity must be considered. For naturalistic observations, there are good statistical methods such as regression models or propensity-score (PS) matching that can be used to control covariates for interfering causal links between intervention and outcomes (17–19). PS matching reduces the number of covariates that need to be controlled for in the outcome model. When appropriately applied, PS matching can produce the same results as obtained by randomized controlled designs (20, 21).

Here we addressed two scientific general goals: (1) to investigate service utilization in routine care by comparing patients' characteristics between the HT sample and the crude inpatient sample and (2) to examine the effectiveness of HT services by comparing treatment outcomes between the HT sample and a matched inpatient sample. For these purposes, a naturalistic observational study-design with PS matching analysis was applied using routinely obtained medical data. We were interested in the following outcomes: treatment duration, reduction of symptoms and increase of functioning, circumstances at discharge, proportion of readmissions, duration until the next readmission, and duration of the readmission period. As HT provides the same range of therapies as the conventional inpatient treatment, we hypothesized that the effectiveness of HT is not inferior to inpatient treatment.

## Materials and methods

### Sample and data collection

The present study included all patients who were treated in either HT or hospital during the study period from June 2016 until December 2017 and meeting the inclusion criteria provided below. We wanted to examine a study period of at least 1 year to compensate for monthly variations including a 6-month follow-up period to calculate the readmission rates. In compliance with the guidelines of the implemented HT services, we set the following inclusion criteria: Patients had to be between 18 and 65 years of age, in need of inpatient treatment, and of a treatment duration of 48 h or more, voluntarily admitted or transferred from inpatient setting voluntarily, and with a permanent residence.

The HT-setting included 201 patients who received 239 treatment episodes. In case when the same patients were treated more than once during the study period, we included only the primary treatment episode (= first case) of each HT patient. Thus, the final sample included *n* = 201 treatment episodes of different HT patients. A treatment episode encompassed the sum of all days treated in HT and if required hospital days directly before, during or after HT.

The control group included in-patients who have been treated only in hospital during the study period in one of the eight wards of the Center for Acute Psychiatric Disorders of the University Hospital of Psychiatry Zurich and who met the above-mentioned inclusion criteria. The sample of the control group included *n* = 1078 in-patients who received 1,459 treatment episodes in hospital. Again, we selected only the primary treatment cases. We also excluded other cases of patients that had been treated both in hospital and in HT during the study period in order to avoid that those cases would get matched with themselves. The crude inpatient sample therefore included *n* = 1078 treatment episodes of different inpatients. The matched inpatient sample was selected from the crude inpatient sample and, consequently, included *n* = 201 inpatients.

We retrieved the data from routine medical data records of the patients. These data have to be collected by federal law in Switzerland (22). Trained physicians who were in charge at the time of the patients' admission and discharge assessed the data. We checked reliability and validity of the data by comparing selected variables with those obtained from other data sources (e.g., accounting systems). The use of such routine data for evaluating health care services has the advantage of providing comprehensive and objective data for every patient (23).

As the data of interest were anonymized prior to access to the study, approval of an institutional review board was not required according to the relevant regulations. The local ethic committee KEK provided a declaration of non-competence for this study.

### Control and outcome measures

#### Control variables

We used the following mentioned control variables to compare the HT sample with the crude inpatient sample and selected them based on (theoretical) associations with both, the assignment to the treatment modality (HT vs. inpatient treatment) and the outcome variables (24). First, we selected sociodemographic variables (gender, age, education, employment status, and Swiss citizenship) and psychiatric diagnoses (main psychiatric diagnosis and number of secondary diagnoses) which were associated with levels of symptoms assessed by the Health of the Nation Outcome Scales (HoNOS) in previous research of the same population (25). Total score on the HoNOS at admission, HoNOS items 1-3 at admission (problems due to harm to others, risk of self-harm, problems related to drug use), GAF score at admission and number of previous hospital stays were used as control variables in line with HT guidelines that emphasize that HT is an alternative for in-patients with severe mental illness, but less suitable for patients at risk for self-harm, risk to others, and problems related to drug use (8–10). The HoNOS range from 1 to 48, with higher scores denoting worse health (26). The GAF scale ranges from 1 to 100 with higher scores indicating better functioning (27).

The following variables were dichotomized and used as matching variables in the PS analysis: gender (male vs. female), education (higher educated [grammar school, higher vocational education, university] vs. other [no education, compulsory education, vocational education, unknown], employment status (employed vs. unemployed), citizenship (Swiss citizenship vs. other), F1 diagnosis (yes vs. no), F3 diagnosis (yes vs. no), secondary diagnoses (present vs. not present), and HoNOS items 1–3 at admission (yes vs. no). Age, HoNOS total score and number of previous hospital stays were used for the PS matching on a continuous scale. Further variables such as F2 diagnosis or GAF scores were not used as matching variables in the final model as they provided no additional value for achieving balanced groups.

#### Outcome variables

We assessed the following outcome variables for both, the HT sample and the matched inpatient sample: Treatment duration, levels of symptoms and functioning, circumstances of discharge, proportion of readmissions, duration until the next readmission, and duration of the readmission period. We calculated the total treatment duration of the HT patients by summing up all treatment days that the patients received in the HT-setting and the inpatient setting (directly before, during, and after HT) of each treatment episode. The following measures were assessed at discharge to determine the severity of symptoms and level of functioning: Health of the Nation Outcome Scales (HoNOS) (26), Global Assessment of Functioning (GAF) scale (27), and Clinical Global Impression (CGI) improvement scale (2–4 = improved, 5 = no change, and 6–8 = worse) (28). In HT patients, HoNOS, GAF and CGI were assessed at admission and at discharge of the entire treatment episode, independent of whether they had been treated in an inpatient setting before or after receiving HT. Missing values on these scales were due to treatment durations < 7 days where scales were not completed (22). Circumstances at discharge were assessed by the proportion of patients who terminated treatment by mutual agreement and the proportion of patients who were able to stay at home after discharge. Treatment duration, duration until the next readmission and duration of the readmission period were measured in whole days. Variables related to readmission were reported for cases treated between 1 June 2016 and 30 June 2017 to ensure a follow-up period of at least 6 months after discharge.

### Statistical analyses

Before PS matching, differences in control variables between the HT sample and the crude inpatient sample were tested using *t*-tests and chi-squared tests for independent samples. Given 201 patients (alpha = 0.05, beta = 0.80), we could expect to find differences of small effects (29). After the PS matching, we used *t*-tests and McNemar-tests for dependent samples. We applied PS matching using a 1:1 ratio with the nearest neighbor matching procedure without replacement. This method is recommended if sample size of the control group is at least 3–4 times larger than that of the treatment group, which was the case in the present study (30). (Other selection procedures, such as optimal matching using 1:2 ratio and caliper matching with a standard deviation-width equal to 0.2 of the logit of the PS produced less balanced samples). We conducted the PS matching analysis following the recommendations of Olmos and Govindasamy (30) and Randolph (31), including a balance check of the matching results and sensitivity analysis of three main outcomes. For the balance check of the matched groups, we performed an omnibus chi-square test (32). In addition, we examined the standardized differences between the two groups for all control variables, which should be smaller than 0.25 (33). We performed a sensitivity analysis based on the method of Rosenbaum to detect biases not identified in the results of non-parametric tests. A gamma value close to 1 indicates that the results are sensitive to bias (34). We used R software (35) with the package matchIt (36) for estimating PS by logistic regression and the packages RItools (37) and matching (38) for balance and sensitivity checks. Other statistical analyses were performed using IBM SPSS, version 23 for Windows (IBM Corp. 2015). Statistical significance was set at the 5% level.

## Results

### Utilization of HT and sample description

Between June 2016 and December 2017, 201 patients received HT, of whom 54.2% were assigned directly to HT and 45.8% were assigned to HT after inpatient treatment in a psychiatric ward. Only 25 (12.4%) patients needed hospital days during or after HT. These patients were more likely to have a substance use disorder (F1 diagnosis) and less likely to have an affective disorder (F3 diagnosis) than HT patients without inpatient days (*p* < 0.05). Direct admissions were 1.5 times more likely to have an affective disorder (F3 diagnosis) and 2.7 times less likely to have a risk of self-harm compared to admissions via psychiatric wards. Table 1 (c.f. column “Inpatients before matching”) shows differences in the control variables between the HT sample and the crude inpatient sample. HT patients were on average older, better educated and characterized by a greater proportion of females and Swiss citizens. In addition, HT patients had more often an affective disorder (F3 diagnosis) and less often a substance use disorder (F1 diagnosis). Patients who received HT did not differ from inpatients with respect to the presence of secondary diagnoses, severity of symptoms and levels of functioning (HoNOS item 1-3, HoNOS total, GAF), and the number of previous hospital stays.

**Table 1:** Sample characteristics of home treatment (HT) patients compared to inpatients before and after propensity score matching during a 19-month period.

	**HT**		**Inpatients before matching**					**Inpatients after matching**				
	***n* = 201**		***n* = 1078**					***n* = 201**				
**Control variables**	**% or Mean**	**SD**	**% or Mean**	**SD**	**Chi-Square or T-value**	***df***	***P*-value**	**% or Mean**	**SD**	**Chi-Square or T-value**	***df***	***P*-value**
Gender, male	34.3		55.3		29.82	1	< 0.001	34.3				1.000
Age at admission	44.4	12.1	39.4	12.9	5.15	1277	< 0.001	44.3	12.5	0.12	200	0.906
**Education**												
Higher educated	35.0^a^		25.5^b^		8.00	2	0.018	33.8		0.56	3	0.906
Unknown	9.0^a^		11.8^a^					10.4				
Fully or partially employed	28.9		31.2		0.43	1	0.514	29.4				1.000
Swiss citizenship	73.1		63.8		6.48	1	0.011	67.2				0.201
**Psychiatric Diagnosis**												
Substance use disorder (F1)	4.0^a^		18.7^b^		36.50	4	< 0.001	3.5		7.04	8	0.532
Psychotic disorder (F2)	31.8^a^		26.0^a^			1		26.4				
Affective disorder (F3)	40.8^a^		27.7^b^			1	< 0.001	40.3				
Neurotic disorder (F4)	15.9^a^		16.7^a^					18.4				
Others	7.5^a^		11.0^a^			1		11.4				
Secondary diagnoses, yes	64.2		61.6		0.48	1	0.488	70.1				0.256
**HoNOS Items 1-3 at Admission**												
Risk of harm to others, yes	28.9		30.1		0.13	1	0.721	27.9				0.911
Risk of self-harm, yes	11.9		17.2		3.41	1	0.065	10.9				0.860
Risk for drugs, yes	28.4		44.8		18.76	1	< 0.001	30.3				0.744
HoNOS total, at admission	19.0	6.7	19.0	6.9	0.03	1275	0.991	18.6	6.6	0.96	200	0.585
GAF, at admission	41.0	14.3	43.4	14.8	2.01	1148	0.045	42.4	14.3	0.79	160	0.430
Previous hospital stays	4.0	6.4	3.7	6.1	1.33	1277	0.184	4.0	7.3	0.09	200	0.932

*For unmatched samples, t-tests for independent samples and Pearson's chi-squared tests were used*.

*For matched samples, t-tests for dependent samples and McNemar tests were used*.

*Identical letters indicate no statistically significant difference by Bonferroni-adjusted chi-squared post-hoc tests at P < 0.05*.

*HoNOS = Health of the Nation Outcome Scales; GAF = Global Assessment of Functioning scale*.

### Goodness of PS matching

**Figure 1:**
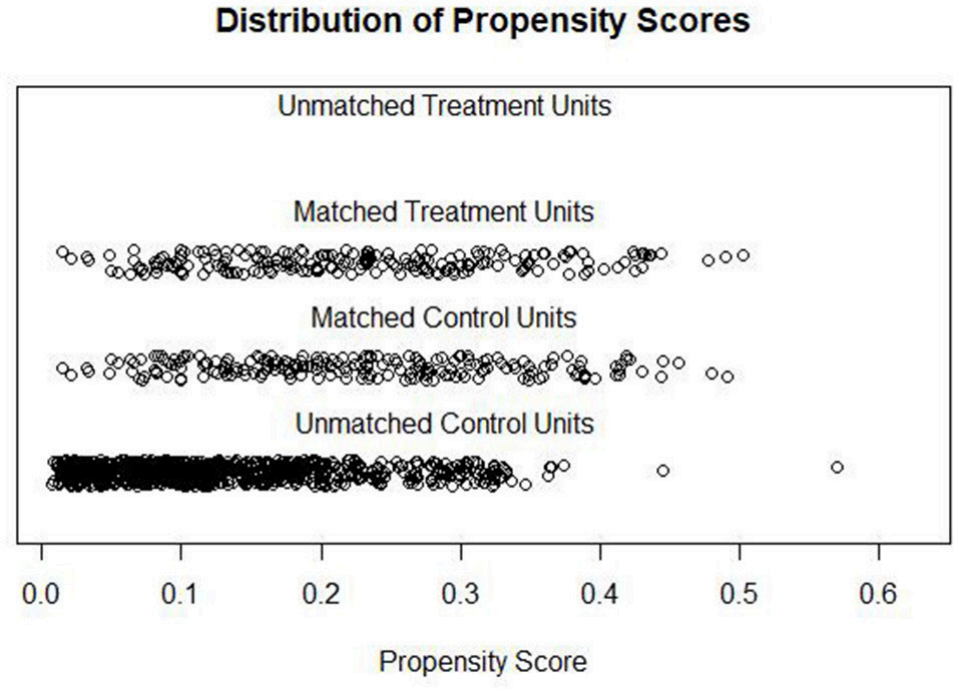
Distribution of Propensity Scores of HT patients (Matched Treatment Units, *n* = 201) and inpatients (Matched Control Units, *n* = 201 and Unmachted Control Units, *n* = 877).

In order to reduce the initial selection bias, the HT sample was 1:1 matched with inpatients selected by PS. Graphical and statistical results of the balance check indicated that PS matching worked very well (c.f. Figure 1). The non-significant chi-square value indicated that the groups were balanced in terms of all control variables (X^2^ = 5.62 (13), *p* = 0.959) (32). The mean distance of all the control variables between the HT and inpatient group was reduced from 0.0828 to 0.0010, while lower quartile-differences indicated better matching (31). Standardized mean differences of all the control variables were small and varied between 0.00 and 0.13 (24). As expected, based on these imbalance indices, the control variables did not significantly differ between the HT sample and the matched inpatient sample (Table 1; c.f. column “Inpatients after matching”).

### Comparison of outcome variables between the matched samples

Details on differences in outcome variables between the two treatment modalities are shown in Tables 2,3. Average treatment duration of the HT patients was 40.5 days and significantly longer than that of the matched inpatient sample (*M* = 26.2). HT patients had significantly fewer hospital days (*M* = 10.3) of which 7.8 days were prior to the patient's transferring to HT. Changes in symptom severity and levels of functioning indicate treatment success in both groups: HoNOS and GAF values were significantly better at discharge in HT patients (HoNOS: *M* = 14.1 [*p* < 0.001]; GAF: M = 67.6 [*p* < 0.001]) and in inpatients (HoNOS: *M* = 15.0 [*p* < 0.001]; GAF: M = 59.0 [*p* < 0.001]) than at admission (HoNOS: *M* = 19.0; GAF: M = 41.0 [HT patients]); HoNOS: *M* = 18.6; GAF: M = 42.4 [inpatients]). At discharge, GAF values were significantly better in the HT sample, but the HoNOS and CGI values did not differ between the two treatment modalities. The majority of patients in both samples were able to stay at home after the end of treatment, with only a small percentage placed in a residential home or referred to another psychiatric or somatic hospital. The proportion of patients who terminated treatment by mutual agreement was significantly higher in HT patients than in inpatients. Further reasons for treatment termination in HT vs. inpatients were the physician's own initiative (4 vs. 6.5%), the patient's own initiative (3.5 vs. 8%), absconding (0.5 vs. 3%), suicide (1 vs. 0.5%), and other reasons not specified (3.5 vs. 3.9%). Table 3 shows that there was no significant difference in the proportion or total number of readmissions, the duration until the next readmission and the duration of the readmission period between the HT patients and the inpatients admitted between June 2016 and June 2017. This was also true when all cases (*n* = 402) were included of the observation period between June 2016 and December 2017. Additionally, the results of the sensitivity analysis indicated that the findings were overall stable with no hidden biases (gamma values for treatment duration, duration until the next readmission and total number of readmissions changed to significance at 1.5 and higher) (34).

**Table 2:** Hospitalization and quality indicators of treatment of matched samples, separated by treatment modality during a 19-month period.

	**HT**		**Inpatients**				
	***n* = 201**		***n* = 201**		**Chi-Square or T-value**		
**Outcome variables**	**% or Mean**	**SD**	**% or Mean**	**SD**		***df***	***P*-value**
**Treatment Duration, Days**							
Total treatment episode	40.5	27.3	26.2	21.8	6.12	200	< 0.001
Home treatment vs. inpatient days	30.2	19.1	26.2	21.8	2.09	200	0.038
Inpatient days	10.3	20.0	26.2	21.8	7.70	200	< 0.001
HoNOS total, at discharge	14.1	6.5	15.0	6.3	1.45	146	0.150
GAF scale, at discharge	67.4	17.8	59.0	16.7	4.79	192	< 0.001
**CGI Improvement Scale, at Discharge**							
Improved	90.5^a^	89.6^a^			1.23	3	0.745
No change	8.0^a^	8.9^a^					
Worse	1.5^a^	1.6^a^					
Mutual agreement of treatment termination	87.5		78.1				0.020
At home after discharge	82.6		78.6				0.382

*T-tests for dependent samples and McNemar tests were used*.

*Identical letters indicate no statistically significant difference by Bonferroni-adjusted Chi-square post-hoc tests at P < 0.05*.

*HoNOS = Health of the Nation Outcome Scales (1-48; higher values = worse); GAF = Global Assessment of Functioning (1-100; higher values = better); CGI = Clinical Global Impression*.

**Table 3:** Readmissions of matched samples of treatment period between 1 June 2016 and 30 June 2017, separated by treatment modality.

	**HT**		**Inpatients**				
	***n* = 103**		***n* = 103**				
**Outcome variables**	**M or %**	**SD**	**M or %**	**SD**	**Chi-Square or T-value**	***df***	***p*-value**
Proportion of readmissions	36.9		35.9				1.000
Number of readmissions	0.9	1.9	1.1	2.2	0.49	102	0.623
Duration until the next readmission, days^a^	295.1	162.7	271.0	160.9	1.09	102	0.279
Duration of the readmission period, days^b^	9.6	21.8	11.1	25.8	0.46	102	0.648

*T-tests for dependent samples and McNemar test were used*.

a *Data are right-censored by end of December 2017*.

b *Duration = 0 of data without readmissions; real duration: M_HT_ = 29.1 days and M_Inpatients_ = 32.6 days*.

## Discussion

This study investigated the utilization and effectiveness of HT by comparing routinely obtained medical data of HT patients with a crude inpatient sample and a 1:1 PS-matched inpatient sample. Naturalistic observations showed that the patients who received HT differed from inpatients receiving conventional treatment in some respects, including sociodemographic variables and psychiatric diagnoses, but not with respect to severity of symptoms and level of functioning at admission. These finding suggests that particularly females and elderly persons with an affective disorder consider HT as a valuable alternative to a conventional inpatient treatment for themselves. Thus, patients' treatment preferences might be also important to consider for HT indication. However, previous research showed that sociodemographic and diagnostic variables were less relevant for treatment success of HT than being uncooperative and at risk of self-harm and harm to others (10). The latter factors did not differ between HT patients and inpatients.

Overall, the results of the PS analysis based on administrative and clinical outcomes indicated that HT seemed to be an effective treatment option for individuals with SAMI as suggested by previous research conducted in other countries (8, 11, 12). PS matching reduced the number of covariates to compare the HT patients with a similar inpatient sample. More precisely, HT was associated with a substantial reduction of hospital days and mean duration of the HT episodes was within the recommended time span of four to six weeks (5). Nevertheless, HT duration was on average 2 weeks longer than the duration of inpatient treatment. In future, HT episodes could possibly be reduced by enabling a smoother and more rapid transition from hospital admission to HT as hospital days before HT accounted for 19.3% of the treatment duration in total. Both HT and inpatient treatment showed the same reduction of symptoms and increase of functioning assessed by the HoNOS (26) and the CGI improvement scale (28). The level of functioning of the HT patients at discharge was even better than that of the inpatients, as assessed by the GAF scale. This is an important finding, because one of the major goals of HT is the provision of practical support in the context of an individual's social and physical environment to stabilize and improve levels of functioning (8, 9). It may also be explicable by the fact, that the HT patients are assessed concerning functioning in their own domestic environment. It can be assumed that patients would show higher levels of functioning compared to the inpatient setting. In addition, the proportion of treatment termination by mutual agreement was higher in HT patients, which might be an indication of treatment satisfaction and a good therapeutic relationship. However, this cannot be confirmed by the present study as no respective scales were applied. In most studies, treatment satisfaction has been reported to be higher for HT services compared to inpatient treatment (12). Furthermore, there were no between-group differences in the outcomes concerning readmissions such as the proportion of readmissions and the duration until the next readmission indicating long-term effectiveness of HT beyond the treatment episodes.

This study was based on routinely obtained medical data, and, thus, specific data such as the patients' satisfaction and the burden of their cohabitants could not be analyzed. Despite the many advantages of routine data in health care service research, another drawback of routine data might be its uncertain quality (23). To address this issue, we carefully checked the reliability and validity of the data by comparing them with other data sources. Based on this assessment, data quality was considered sufficiently high. A further limitation of the study might be its observational study design for drawing causal conclusions. For instance, the uniqueness of the multiprofessional HT team may have influenced the results. In contrast to randomized controlled designs, the outcome assessment was non-blinded. However, sophisticated PS matching is considered a good method to remove selection bias in observational studies when randomized controlled designs are not feasible, potential confounders are known, and the sample size of the control group is sufficiently large (30). In this study, the sample size of potential control cases was very large, and the balance check pointed to successful PS matching. The sensitivity analysis further suggested that the results are robust. All data were collected from only one hospital in Switzerland, thus, results may not be generalized for the whole country. However, the examined HT services are included in one of the largest psychiatric hospitals in Switzerland with an urban and periurban catchment area.

In conclusion, HT seems to be an effective alternative to inpatient treatment also in a Swiss urban region for a wide range of patients with SAMI concerning the reduction of hospital days, the improvement of symptoms and functioning and readmission rates. Although HT reduces the need for inpatient stays, it cannot fully replace hospital admissions in all cases. Besides medical aspects, some groups of patients (e.g., women, patients with affective disorders) might be particularly interested in HT services. Further studies are needed to examine other aspects of effectiveness, for instance including cost-effectiveness, patients' satisfaction and treatment interventions of HT services.

## Author contributions

SM, HS, and MJ contributed to the conception and design of the study. SM and DS organized the database and performed statistical analysis. SM wrote the first draft of the manuscript. SM, HS, MJ, DS, and ES contributed to the manuscript revision and approved the submitted version.

### Conflict of interest statement

The authors declare that the research was conducted in the absence of any commercial or financial relationships that could be construed as a potential conflict of interest.
